# Prevalence of cryptococcal antigen (CrAg) among HIV-positive patients in Eswatini, 2014–2015

**DOI:** 10.4102/ajlm.v9i1.933

**Published:** 2020-07-29

**Authors:** Samson M. Haumba, Mitsuru Toda, Rossana Jeffries, Peter Ehrenkranz, Munyaradzi Pasipamire, Trong Ao, Nomthandazo Lukhele, Sikhathele Mazibuko, Mandzisi Mkhontfo, Rachel M. Smith, Tom Chiller

**Affiliations:** 1 University Research Co., LLC, Mbabane, Eswatini; 2 Epidemic Intelligence Service (EIS), Division of Scientific Education and Professional Development, Center for Surveillance, Epidemiology, and Laboratory Services (CSELS), Centers for Disease Control and Prevention (CDC), Atlanta, Georgia, United States; 3 Mycotic Diseases Branch (MDB), Division of Foodborne, Waterborne, and Environmental Disease (DFWED), National Center for Emerging and Zoonotic Infectious Diseases (NCEZID), Centers for Disease Control and Prevention (CDC), Atlanta, Georgia, United States; 4 Global Development, Bill and Melinda Gates Foundation, Seattle, Washington, United States; 5 Ministry of Health Eswatini National AIDS Programme (ENAP), Mbabane, Eswatini; 6 Centers for Disease Control and Prevention (CDC), Mbabane, Eswatini

**Keywords:** cryptococcal antigenaemia screening, prevalence, people living with HIV, cryptococcal meningitis, advanced HIV disease package, Eswatini

## Abstract

**Background:**

Cryptococcal meningitis is a leading cause of death amongst people living with HIV. However, routine cryptococcal antigen (CrAg) screening was not in the national guidelines in Eswatini.

**Objectives:**

A cross-sectional study was conducted between August 2014 and March 2015 to examine CrAg prevalence at Mbabane Government Hospital in Eswatini.

**Methods:**

We collected urine and whole blood from antiretroviral-therapy-naïve patients with HIV and a cluster of differentiation 4 (CD4) counts < 200 cells/mm^3^ for plasma and urine CrAg lateral flow assay (LFA) screening at the national HIV reference laboratory. Two CD4 cut-off points were used to estimate CrAg prevalence: CD4 < 100 and < 200 cells/mm^3^. Sensitivity and specificity of urine CrAg LFA was compared to plasma CrAg LFA.

**Results:**

Plasma CrAg prevalence was 4% (8/182, 95% confidence interval [CI]: 2–8) amongst patients with CD4 counts of < 200 cells/mm^3^, and 8% (8/102, 95% CI: 3–15) amongst patients with CD4 counts of < 100 cells/mm^3^. Urine CrAg LFA had a sensitivity of 100% (95% CI: 59–100) and a specificity of 80% (95% CI: 72–86) compared with plasma CrAg LFA tests for patients with CD4 < 200 cells/mm^3^. Forty-three per cent of 99 patients with CD4 < 100 were at World Health Organization clinical stages I or II.

**Conclusion:**

The prevalence of CrAg in Eswatini was higher than the current global estimate of 6% amongst HIV-positive people with CD4 < 100 cell/mm^3^, indicating the importance of initiating a national screening programme. Mechanisms for CrAg testing, training, reporting, and drug and commodity supply issues are important considerations before national implementation.

## Introduction

Cryptococcosis, caused by *Cryptococcus neoformans* or *Cryptococcus gattii*, is an invasive and life-threatening fungal infection, often affecting immunocompromised patients. Cryptococcal meningitis, a type of cryptococcosis affecting the brain meninges, is one of the leading opportunistic infections and causes of death amongst people living with HIV. It especially affects patients with advanced HIV who have a cluster of differentiation 4 (CD4) count < 200 cells/mm^3^ or who are at stages III or IV of the World Health Organization (WHO) HIV infection clinical stages.^1^ In 2014, globally, the cryptococcal antigenaemia prevalence was estimated at 6% (278 000) amongst patients with CD4 < 100 cells/mm^3^, with approximately 223 100 cryptococcal meningitis cases occurring annually.^2^ In the same year, 2014, the annual deaths due to cryptococcal meningitis were estimated at 181 100, with 75% (135 900) of deaths occurring in sub-Saharan Africa.^2^

The WHO recommends targeted screening of HIV-positive patients to enable early detection and pre-emptive treatment of cryptococcal infection.^1^ Pre-emptive antifungal therapy can prevent cryptococcal meningitis-related mortality and morbidity in cryptococcal antigen (CrAg)-positive antiretroviral therapy (ART)-naïve patients.^3^ The CrAg lateral flow assay (LFA) is an affordable,^4^ cost-effective,^5^ and simple point-of-care assay for CrAg testing in blood and cerebrospinal fluid (CSF). CrAg can be detected a median of three weeks before clinical evidence of cryptococcal meningitis.^6^

The prevalence of HIV in Eswatini (formerly Swaziland) is amongst the highest in the world.^7^ Despite the high HIV prevalence and known risk of cryptococcal meningitis, CrAg prevalence amongst people with advanced HIV disease, or CD4 < 200 cells/mm^3^, is unknown in Eswatini. At the time of this study, routine CrAg screening was not included in the national guidelines and its clinical utility was unknown. We conducted a cross-sectional study to examine CrAg prevalence at a national hospital in Eswatini. We also compared sensitivity and specificity of urine CrAg LFA relative to plasma CrAg LFA.

## Methods

### Ethical considerations

The Swaziland Scientific and Ethics Committee (MH/599C/ FWA 000 15267), and the Centers for Disease Control and Prevention Institutional Review Board reviewed and approved the study protocol (CGH HSR tracking #2014-139). Study nurses obtained verbal informed consent from all participants.

### Study location and population

A cross-sectional study was implemented at Mbabane Government Hospital, a national hospital, in Eswatini. Mbabane Government Hospital is a public hospital with a 500 bed capacity. It is the largest HIV treatment and care centre in Eswatini and serves the population of the Hhohho region (320 651), as well as the population of the nation at large (1 093 2381 million).^8^

We assessed eligibility of ART-naïve adults ≥ 18 years old in the study who attended the Voluntary Counseling and Testing clinic, as well as hospitalised patients, by determining their CD4 levels using Alere Pima^TM^ (Abbott Laboratories, Chicago, Illinois, United States). For HIV-positive patients with CD4 ≤ 350 cells/mm^3^ by the Alere Pima^TM^ CD4 test, a confirmatory test was completed using BD FACSCalibur^TM^ (Beckton Dickinson, San Jose, California, United States) flow cytometry. Patients with CD4 < 200 cells/mm^3^ on BD FACSCalibur^TM^ were enrolled in the study.

Enrolment occurred from 18 August 2014 to 19 March 2015. We excluded pregnant women, patients with a previous diagnosis or treatment for cryptococcal meningitis, and patients who had ever received fluconazole for < 5 days before study enrolment. Blood and urine samples were obtained during the patient’s first visit to the HIV testing clinic or soon after HIV testing, if an inpatient. Plasma and urine CrAg screening were conducted using a LFA (IMMY, Norman, Oklahoma, United States) at the hospital laboratory for patients both with and without signs of cryptococcal meningitis.

### Data collection

Trained study nurses and research assistants conducted data collection. Study nurses and research assistants gave a communication leaflet providing basic information on *Cryptococcus* spp. and HIV infection as well as the objectives of the study to eligible patients. After the informed consent process, data on patient demographic characteristics including age, sex, marital status, area of residence (rural or urban), level of education, and the clinical stage of HIV infection^9^ were collected.

### Clinical procedures

At enrolment, study nurses assessed patients’ clinical signs and symptoms of cryptococcal meningitis such as fever, headache, neck stiffness, altered mental status, photophobia, nausea, night sweats, cough, vomiting, shortness of breath, skin papules, or kerning sign. Patients with positive plasma CrAg results and signs and symptoms of cryptococcal meningitis underwent a lumbar puncture as part of routine practice. CSF samples obtained from the lumbar puncture were collected for CrAg LFA to diagnose cryptococcal meningitis. Patients diagnosed with cryptococcal meningitis were treated according to a standard of care based on WHO guidelines,^3^ which included amphotericin B (0.7 mg/kg/day) and fluconazole (800 mg) for 2 weeks followed by 400 mg of fluconazole for 8 weeks and then maintained at 200 mg for secondary prophylaxis. Patients who refused a lumbar puncture but had positive plasma CrAg results were offered pre-emptive therapy which followed WHO recommendations of fluconazole 800 mg/day for the first 2 weeks, followed by 8 weeks of 400 mg/day of fluconazole daily, and a 200 mg/day fluconazole maintenance dosage.^3^

The South African clinical guidelines (2013) were followed for patients with positive plasma CrAg and negative CSF CrAg results.^10^ ART was delayed for 2 weeks to decrease the risk of immune reconstitution inflammatory syndrome, and they were prescribed pre-emptive fluconazole oral treatment to prevent the development of meningeal infection.^10^ Because of resource constraints, patients’ cryptococcal infections were not verified using X-ray or other imaging technology, and we did not follow the individual patients’ clinical course to collect outcome measures for the purposes of this study.

### Laboratory procedures

All blood and urine specimens were packaged according to WHO standards. Study nurses collected fresh urine samples and recorded urine CrAg LFA positive results. In addition, study nurses collected whole blood in ethylene diamine tetra acetic acid-treated test tubes and stored it at room temperature. The blood was centrifuged for 5 min at 300 revolutions per minute to obtain plasma samples on the same day that blood was collected. Plasma and urine samples were stored at 2 °C – 8 °C for up to 72 h. CD4 retesting was completed using FACSCalibur^TM^ and research assistants conducted CrAg testing at the laboratory the next day using CrAg LFA on samples from patients with CD4 < 200 cells/mm^3^. In addition, research assistants performed daily positive and negative control LFA testing as well as lot-to-lot testing to ensure the quality of reagents. Research assistants communicated all laboratory results to physicians for clinical management, and clinicians informed the participants of their results.

### Sensitivity and specificity analyses

We compared urine and plasma CrAg LFA results using plasma LFA as the gold standard. Records were retained for study purposes and not for clinical diagnosis or treatment purposes, and our specificity and sensitivity analyses did not take into account clinical or radiological findings.

### Analyses

Data were double entered using EpiData (EpiData Association, Odense, Denmark). The descriptive analyses and calculation of Wilson scores on 95% confidence intervals (CI) on the CrAg prevalence were conducted using Stata 15 (StataCorp, College Station, Texas, United States).

## Results

**FIGURE 1:**
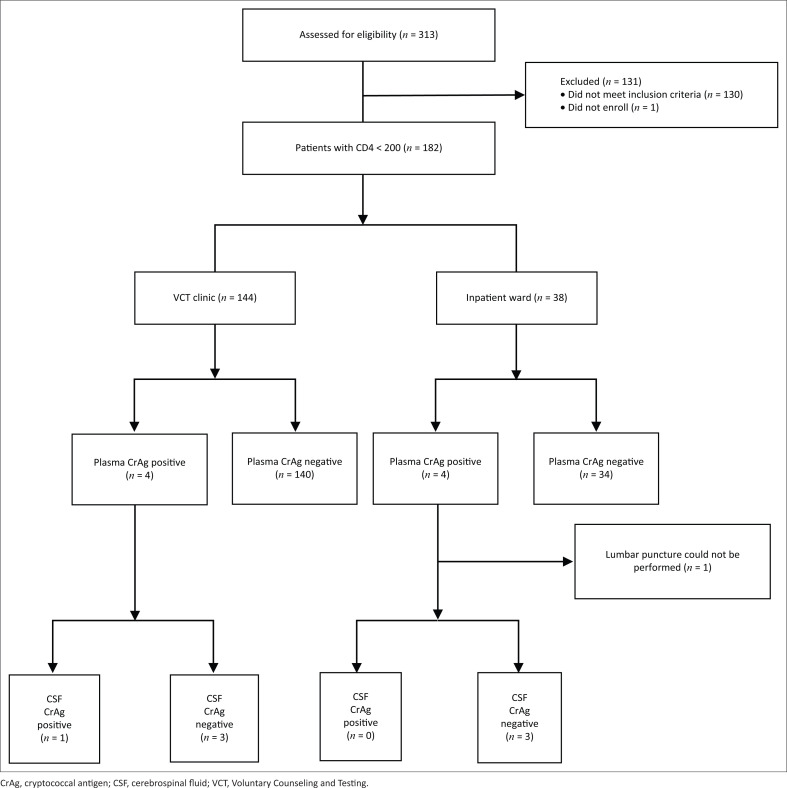
Study flow for cryptococcal screening at Mbabane Government Hospital, Eswatini, August 2014 to March 2015.

Of 313 patients initially screened for eligibility, 58% (182/313) had CD4 counts < 200 cells/mm^3^ by FACSCalibur^TM^ (Figure 1). Of the 182 eligible patients, 79% (144/182) were from the outpatient Voluntary Counseling and Testing clinic and 21% (38/182) were from the inpatient ward (Table 1).

**TABLE 1:** Characteristics of patients enrolled in the CrAg screening study of patients with a CD4 count < 200 cells/mm^3^, Eswatini, August 2014 to March 2015.

Variable	*N* = 182	%
**CD4 count**		
< 100 cells/mm^3^	102	56
100 cells/mm^3^ – 199 cells/mm^3^	80	44
**Patient recruitment site**		
VCT clinic	144	79
In-patient ward	38	21
**Sex**		
Male	106	58
Female	76	42
Age, median [IQR]*	35	30–42
**Marital status**		
Single	113	62
Married	61	34
Widowed, divorced, or separated	8	4
**Education**		
No schooling or did not complete primary	86	47
Completed primary	75	41
Completed secondary or higher	21	12
**Residence type**		
Urban	111	61
Rural	71	39
**Region of residence**		
Hhohho	156	86
Manzini	18	10
Shiselweni	5	3
Lubombo	3	2
**WHO clinical disease stage**		
Stage I or II	99	54
Stage III	73	40
Stage IV	10	5

CrAg, cryptococcal antigen; IQR, interquartile range; VCT, Voluntary Counseling and Testing; WHO, World Health Organization.

* Age, *N* = 181 (age information was missing for one patient).

Of the 182 patients with CD4 counts < 200 cells/mm^3^ by FACSCalibur^TM^, 42% (76/182) were women, the median age of the participants was 35 years (interquartile range: 30–42)], and 61% (111/182) were from urban areas (Table 1). Most (86%, 156/182) patients were from the Hhohho region, 10% (18/182) from Manzini, 3% (5/189) from Shiselweni, and 2% (3/182) from Lubombo. Almost half of the patients (47%, 86/182) did not complete primary school or did not receive any schooling, 41% (75/182) completed primary school, and 12% (21/182) completed secondary school or higher. Most (62%, 113/182) patients were single and never married, 34% (61/182) were married, and 4% (8/182) were widowed, divorced, or separated. More than half of the patients (54%, 99/182) were at WHO clinical stages I or II, 40% (73/182) at stage III, and 5% (10/182) at stage IV.

Of the 182 patients with CD4 count < 200 cells/mm^3^, 56% (102/182) had a CD4 count < 100 cells/mm^3^. Forty-three percent (44/102) of these were at WHO clinical stages I or II and 57% (58/102) at stages III or IV. Of patients with CD4 counts 100 cells/mm^3^ – 199 cells/mm^3^, 69% (55/80) were at WHO clinical stages I or II and 31% (25/80) at stages III or IV.

### Cryptococcal antigen lateral prevalence

The plasma CrAg prevalence was 8% (8/102, 95% CI: 3–15) amongst patients with CD4 counts < 100 cells/mm^3^. When considering the higher cut-off point of a CD4 count < 200 cells/mm^3^, the plasma CrAg prevalence decreased to 4% (8/182, 95% CI: 2–8) (Figure 1). All eight plasma CrAg-positive patients had CD4 cell counts < 100 cells/mm^3^ and none had CD4 cell counts in the 100 cells/mm^3^ – 199 cells/mm^3^ range (prevalence 4% [8/182], 95% CI: 2–8).

Of the patients at the Voluntary Counseling and Testing clinic, 47% (68/144) had CD4 counts 100 cells/mm^3^ – 199 cells/mm^3^, and 53% (76/144) patients had CD4 counts < 100 cells/mm^3^. Of the 76, 5% (4/76), were plasma CrAg-positive. Three out of the four patients who were plasma CrAg-positive were CSF CrAg negative; one patient did not complete a lumbar puncture.

Of the patients at the inpatient ward, 32% (12/38) had CD4 counts 100 cells/mm^3^ – 199 cells/mm^3^ and 68% (26/38) had CD4 counts < 100 cells/mm^3^. Fifteen percent (4/26) of patients, all with CD4 counts < 100 cells/mm^3^, were plasma CrAg-positive. Of the four patients who were plasma CrAg-positive at the inpatient ward, one patient was CSF CrAg-positive and the other three patients were CSF CrAg negative (Figure 1).

### Sensitivity and specificity of urine cryptococcal antigen lateral flow assay

Urine samples were collected from 168 patients; 82% (137/168) from the Voluntary Counseling and Testing clinic and 18% (31/168) from the inpatient ward. Forty-six percent (77/168) of patients had CD4 counts of 100 cells/mm^3^ – 199 cells/mm^3^ and 54% (91/168) had CD4 counts < 100 cells/mm^3^ by FACSCalibur^TM^. Urine CrAg-positive prevalence was 25% (23/91) amongst patients with CD4 counts < 100 cells/mm^3^ and 22% (17/77) amongst patients with CD4 counts 100 cells/mm^3^ – 199 cells/mm^3^.

Urine CrAg LFA had a sensitivity of 100% (95% CI: 59–100) and a specificity of 80% (95% CI: 72–86) compared with plasma CrAg LFA for patients with CD4 < 200 cells/mm^3^. There were seven true positives, 33 false positives (i.e., positive on urine CrAg LFA but negative on plasma CrAg LFA), 128 true negatives, and zero false negatives (i.e., negatives on urine CrAg LFA but positive on plasma CrAg LFA) (Table 2).

**TABLE 2:** Sensitivity and specificity of urine cryptococcal antigen lateral flow assay compared with plasma cryptococcal antigen lateral flow assay for patients with a CD4 count < 200 cells/mm^3^, Eswatini, August 2014 to March 2015.

Urine CrAg LFA	Plasma CrAg LFA		
	Positive	Negative	Total
Positive	7	33	40
Negative	0	128	128

**Total**	**7**	**161**	**168**

CrAg, cryptococcal antigen; LFA, lateral flow assay.

Sensitivity: 100% (95% CI: 59–100).

Specificity: 80% (95% CI: 72–86).

For patients with < 100 cells/mm^3^, sensitivity of the urine CrAg LFA compared with plasma CrAg LFA was 100% (95% CI: 59–100), and specificity was 81% (95% CI: 71–89). There were seven true positives, 16 false positives (i.e., positive on urine CrAg LFA but negative on plasma CrAg LFA), 68 true negatives, and zero false negatives (i.e., negatives on urine CrAg LFA but positive on plasma CrAg LFA) (Table 3).

**TABLE 3:** Sensitivity and specificity of urine cryptococcal antigen lateral flow assay compared with the plasma cryptococcal antigen lateral flow assay for patients with a CD4 count < 100 cells/mm^3^, Eswatini, August 2014 to March 2015.

Urine CrAg LFA	Plasma CrAg LFA		
	Positive	Negative	Total
Positive	7	16	23
Negative	0	68	68

**Total**	**7**	**84**	**91**

CrAg, Cryptococcal antigen; LFA, Lateral flow assay.

Sensitivity: 100% (95% CI: 59–100).

Specificity: 81% (95% CI: 71–89).

## Discussion

We conducted a cross-sectional study on CrAg prevalence at a national reference hospital in Eswatini. The study showed that patients with CD4 < 100 cells/mm^3^ at the national reference hospital had a CrAg prevalence of 8%, which is higher than the 2014 global estimate of 6%^2^ and similar to the prevalence found in Uganda^5^ and South Africa.^11^ This study is relevant in the context of recent WHO guidelines on managing advanced HIV disease, rapid initiation of ART,^1,9^ and the current recommendation to include CrAg screening in national guidelines in Eswatini^12^ and in other countries.^13^

Combining CrAg screening and early treatment for cryptococcal infection are cost-effective interventions compared with standard care to prevent morbidity and mortality from cryptococcal meningitis amongst immunocompromised people living with HIV.^5,11,14,15,16,17^ Most importantly, CrAg screening has the potential to improve HIV outcomes by reducing morbidity and mortality. The ‘Reduction of Early Mortality among HIV-infected Subjects sTarting AntiRetroviral Therapy’ (REMSTART) trial in Tanzania and Zambia showed CrAg screening, pre-emptive treatment, and community support that led to a 28% reduction in mortality amongst patients with advanced HIV disease,^18^ and the ‘Reduction of EArly mortaLITY in HIV-infected African adults and children starting antiretroviral therapy’ (REALITY) trial in Kenya, Malawi, Uganda, and Zimbabwe showed that an enhanced prophylaxis package reduced mortality by 27%.^19^

CD4 cell counts are essential in determining the timing of ART initiation for patients with advanced HIV disease, because early ART initiation could lead to worse outcomes for CrAg-positive patients.^20^ Current WHO recommendations include CrAg screening for people living with HIV at CD4 cell counts < 100 cells/mm^3^, and offering pre-emptive antifungal therapy before initiating ART.^1^ In the era of ‘90-90-90’^21^ and ‘test and start’ strategies where rapid ART initiation is promoted, there is potential risk in offering ART to patients presenting with advanced HIV disease with low CD4 cell counts or WHO clinical stages III or IV^22^ who are at high risk of opportunistic infection.^23^ Screening these patients for undiagnosed Cryptococcus infection can prevent life-threatening immune reconstitution inflammatory syndrome.^26^ Yet, assessment of a clinical stage without a CD4 count is not sufficient. In our study, 43% of patients with CD4 < 100 cells/mm^3^ were classified as WHO clinical stages I or II. These patients would not have been screened for CrAg in the absence of CD4 cell count testing.

Although studies suggest that screening may be considered at CD4 < 200 cells/mm^3^,^24^ our study suggests that selecting for patients with CD4 < 100 cells/mm^3^ might be a priority, since all plasma CrAg-positive patients had CD4 < 100 cells/mm^3^. Since CrAg-positive patients were found at both outpatient (prevalence: 5%) and inpatient (prevalence: 15%) facilities, CD4-directed CrAg screening should be recommended for patients presenting in either setting.

The sensitivity and specificity of urine CrAg compared with plasma CrAg testing were similar to the finding of a Tanzanian study.^25^ Those findings showed that urine CrAg testing could be useful in ruling out disease, if the result is negative, but may not be a reliable stand-alone screening tool because of the prevalence of false-positive results.

The Eswatini Ministry of Health revised its HIV guidelines in 2018 and included CrAg screening for adults with CD4 cell < 100 cells/mm^3^ or children with CD4 counts at less than 25% of total lymphocytes,^12^ as well as pre-emptive treatment as a package for advanced disease management to reduce morbity and mortality.^26,27,28^ Education of patients is important in order to inform them that cryptococcal meningitis is not, as some Swazi patients refer to it, the ‘headache that kills’, but rather a preventable and treatable disease (personal communication with patients). Before rollout, training of the clinical and laboratory workforce on CrAg testing and reporting, and ensuring adequate supplies of antifungal prophylaxis, treatment (fluconazole, flucytocine, and amphotericin B), lumbar puncture kits and CrAg LFA kits are essential. Moreover, joint ownership of the CrAg screening program by the Swaziland National HIV/AIDS Programme and Swaziland Health Laboratory Services have been established in-country, which could help accelerate buy-in from the clinical and laboratory workforces and successfully roll out the national CrAg screening programme.

### Limitations

There are several limitations to this study. Firstly, the study was conducted in one hospital, thus it is not a nationally-representative sample. However, this hospital is a national reference hospital that captures a large number of patients who are CrAg-positive. Secondly, the study only assessed ART-naïve patients, whereas patients failing ART may also have CD4 cell counts < 100 cell/mm^3^ and become at risk of cryptococcal infection. In addition, the cross-sectional study design and small sample size limited our ability to measure or compare patient outcomes. Further studies to examine patient treatment and outcome measures, and address CrAg prevalence among ART-experienced patients, are warranted. Finally, sensitivity and specificity analyses of urine CrAg LFA were evaluated compared with plasma LFA and did not take into account radiological or clinical findings.

### Conclusion

This study indicated that the CrAg prevalence at a national hospital in Eswatini was higher than the global CrAg estimates of 6% amongst HIV-positive people with CD4 < 100 cell/mm^3^. Our findings support the current Eswatini national cryptococcal screening and treatment guidelines. Issues of drug and commodity supply and training mechanisms for CrAg testing, monitoring, evaluation and reporting are important considerations, before national implementation of plasma CrAg LFA screening.

### Trustworthiness

The following measures were undertaken to ensure trustworthiness:

Cryptococcal antigen lateral flow assay Quality Control: Laboratory technologists at the National Reference Laboratory conducted quality control testing for the CrAg LFA reagents on a weekly basis. A positive and negative control LFA test as well as lot-to-lot testing were performed to ensure the quality of the reagents. The reagents were discarded when the positive or negative controls did not yield the expected results, and a new set of LFA reagents were requested from the laboratory supervisor and tested prior to further patient testing for CrAg.

Training of clinicians and study personnel: The CDC-Eswatini and CDC-Atlanta study teams, who were experienced in CrAg screening, trained the research assistants, clinicians and laboratory personnel involved in the care of patients.

The training of clinicians and study personnel covered the following topics:

- Basic information about cryptococcal disease, including early and disseminated infection
- Explanation of the rationale behind the implementation of this screening programme
- Review of the algorithm for implementation and associated study forms and documents
- Review of procedure for sending samples to the National Reference Laboratory and obtaining results
- Role-playing using different clinical scenarios in order to ensure proper adherence to the algorithm and flow of patients
- Instructions and practice in performing and interpreting the LFA CrAg test
- Review of the procedure for sending CSF CrAg results to HIV Care Centre staff
- Reviewing standard operating procedures relevant to the laboratory staff
- Study protocol
- Data collection tools
- Research ethics
- Quality Assurance.
